# Micro-structural white matter abnormalities in new daily persistent headache: a DTI study using TBSS analysis

**DOI:** 10.1186/s10194-023-01620-2

**Published:** 2023-07-02

**Authors:** Yanliang Mei, Wei Wang, Dong Qiu, Ziyu Yuan, Xiaoyan Bai, Hefei Tang, Peng Zhang, Xue Zhang, Yaqing Zhang, Xueying Yu, Binbin Sui, Yonggang Wang

**Affiliations:** 1 Headache Center, Department of Neurology, Beijing Tiantan Hospital, Capital Medical University, No.119 South Fourth Ring West Road, Fengtai District, Beijing, 100070 China; 2 Tiantan Neuroimaging Center of Excellence, China National Clinical Research Center for Neurological Diseases, No.119 South Fourth Ring West Road, Fengtai District, Beijing, 100070 China; 3 Department of Radiology, Beijing Tiantan Hospital, Capital Medical University, Beijing, Neurosurgical Institute, Beijing, China

**Keywords:** Tract-based spatial statistics, New daily persistent headache, Diffusion tensor imaging, Micro-structural abnormalities, Pain

## Abstract

**Background:**

New daily persistent headache (NDPH) is a rare primary headache disorder characterized by daily and persistent sudden onset headaches. The pathogenesis of NDPH remains unclear, and there are few white matter imaging studies related to NDPH. The purpose of this study was to investigate the micro-structural abnormalities of white matter in NDPH and provided insights into the pathogenesis of this disease based on tract-based spatial statistics (TBSS).

**Methods:**

Twenty-one patients with NDPH and 25 healthy controls (HCs) were included in this study. T1 structural and diffusion magnetic resonance imaging (MRI) were acquired from all participants. Differences in the fractional anisotropy (FA), mean diffusivity (MD), axial diffusivity (AD), and radial diffusivity (RD) between patients with NDPH and HCs were investigated using TBSS analysis.

**Results:**

Significantly decreased FA, increased MD and RD were found in patients with NDPH compared to HCs. White matter regions overlaid with decreased FA, increased MD and RD were found in 16 white matter tracts from the Johns Hopkins University ICBM-DTI-81 White-Matter Atlas and Johns Hopkins University White-Matter Tractography Atlas. Specifically, these white matter regions included the right anterior thalamic radiation (ATR), body of the corpus callosum (BCC), bilateral cingulum, left hippocampal cingulum (CGH), left corticospinal tract (CST), forceps major, fornix, left inferior fronto-occipital fasciculus (IFOF), bilateral inferior longitudinal fasciculus (ILF), left posterior limb of the internal capsule (PLIC), right retrolenticular part of the internal capsule (RPIC), splenium of the corpus callosum (SCC), right superior longitudinal fasciculus (SLF) and left uncinate fasciculus (UF). After Bonferroni correction, there were no correlations between the FA, MD, AD and RD values and the clinical characteristics of patients with NDPH (*p* > 0.05/96).

**Conclusion:**

The results of our research indicated that patients with NDPH might have widespread abnormalities in the white matter of the brain.

## Background

New daily persistent headache (NDPH) is a rare headache disorder characterized by daily headaches that become unremitting soon after onset, typically in individuals with no previous history of headache [1, 2]. Patients with NDPH can recall and accurately describe the exact time of headache onset [3]. According to previous epidemiological studies, the prevalence of NDPH is 0.03 to 0.1% in the general population [4, 5]. The underlying pathogenesis of NDPH remains unclear [6]. In recent years, neuroimaging studies have been increasingly applied to primary headache [6–10] showing, specifically in migraine, widespread white matter abnormalities [11–14]. Contrariwise, there have been relatively few neuroimaging studies of NDPH, suggesting a lack of association with infarct-like lesions or white matter abnormalities in these patients investigated by conventional magnetic resonance imaging (MRI) [15]. However, no relevant research has been carried out to determine whether there are changes in the white matter micro-structure in patients with NDPH.

The diffusion tensor imaging (DTI) is an MRI-based technique that measures the micro-structural integrity of white matter fiber bundles, making it an effective tool able to identify subtle tissue changes that affect the structural connectivity integrity of the brain and the interregional transmission of information [16, 17]. The four main metrics evaluated using DTI are fractional anisotropy (FA), mean diffusivity (MD), radial diffusivity (RD), and axial diffusivity (AD) [18]. FA measures the directionality and coherence of water diffusion, reflecting fiber density, axonal diameter, and myelination in white matter. MD measures the average molecular motion, or diffusion, within a voxel, and it can increase with tissue damage, reflecting a potential decrease in barriers to diffusion. RD and AD are measures of diffusivity perpendicular and parallel to the fibers, respectively, providing insights into myelin and axon integrity [19]. The tract-based spatial statistics (TBSS) is a spatial statistical analysis method based on the white matter skeleton [20]. By constructing an average FA skeleton map of the images and projecting all FA of the subjects onto this skeleton map, the FA values of each voxel on the skeleton map is very close to the FA values of the nearest white matter fiber bundle center. TBSS can fully reflect the subtle structural changes in white matter, which has become an accurate and widely used method for exploring white matter fiber tracts [21].

DTI data analysis technology based on TBSS has been widely used in central nervous system diseases in the past decades, and can effectively assess the micro-structural integrity of white matter [16, 22–24]. In this study, we hypothesized that: 1) patients with NDPH have significant white matter micro-structure changes compared with healthy controls (HCs); 2) diffusivity metrics of the white matter are significantly correlated with disease characteristics in patients with NDPH.

## Methods

### Participants

An observational study with a cross-sectional design was conducted. Fifty-four participants, including 24 patients with NDPH and 30 HCs, were consecutively enrolled. Between October 2020 and October 2022, a total of 24 patients with NDPH were recruited from the headache outpatient unit at Beijing Tiantan Hospital (Capital Medical University). The inclusion criteria for patients with NDPH were as follows: 1) NDPH diagnosis based on the International Classification of Headache Diseases, 3rd edition (ICHD-3) [1]; 2) 14–60 years of age; 3) feasibility of MRI scan; 4) without preventive treatment for at least 3 months; and 5) without the history of excessive use of acute treatment drugs. The general exclusion criteria for patients with NDPH and HCs were as follows: 1) combined with other types of primary headache and pain disorders; 2) pregnancy or breastfeeding; 3) combined with other neurological, cardio-cerebrovascular, and endocrine system diseases; 4) any drug or alcohol abuse history; 5) first degree relative with headaches;6) poor quality of MRI data (significant susceptibility artifact or incomplete raw MRI data); and 7) significant brain lesions or white matter hyperintensities (Fazekas score > 1, especially at the level of the lateral ventricular body).

### Demographic data and neuropsychological tests

Demographics, body mass index (BMI), headache disease duration (years), Visual Analogue Scale (VAS), Patient Health Questionnaire-9 (PHQ-9) scores, Headache Impact Test-6 (HIT-6) scores, Generalized Anxiety Disorder-7 (GAD-7) scores and Pittsburgh Sleep Quality Index (PSQI) scores were collected in patients with NDPH. Anxiety and depression symptoms were measured by the PHQ-9 and the GAD-7, respectively. The PSQI was a useful tool to evaluate the quality and patterns of sleep. The HIT-6 was also used to assess impact intensity of headache. The score of PHQ-9 was commonly used to screen for depression with a recommended cut-off score of 10 [25]. The scores of 10 or higher on the GAD-7 indicated generalized anxiety disorder [26]. The PSQI score ≥ 7 was defined as poor quality of sleep [27].

This was a sub-study of the ongoing China HeadAche DIsorders RegiStry Study (CHAIRS, unique identifier: NCT05334927) and was approved by the local ethics committee of Beijing Tiantan Hospital, Capital Medical University (number: KY2022-044). Informed consent was obtained from each participant before their participation, in accordance with the principles of the Declaration of Helsinki.

### MRI acquisition

The GE 3.0 Tesla MR scanner (Signa Premier, GE Healthcare) with a 48-channel head coil was used at the National Neurological Center of Beijing Tiantan Hospital to acquire 3D T1 structural and diffusion MRI data. The participants were instructed to remain motionless with their eyes closed during the MRI acquisition. T1 structural images were acquired using the following parameters: MP-RAGE sequence, preparation time = 880 ms, recovery time = 400 ms, acceleration factor = 2, acquisition time = 4:00, field of view = 250 × 250 mm^2^, flip angle = 8°, slices = 192, and 1 × 1 × 1mm^3^ of spatial resolution. The following DTI parameters were used: repetition time = 5285 ms, echo time = 85 ms, data matrix = 104 × 104, field of view = 208 × 208 mm^2^, slice thickness = 2 mm, slices = 78, gradient direction = 108, and diffusion sensitivity coefficients (b) = 0, 1000, and 2000s/mm^2^.

### MRI data processing and analysis

Before pre-processing, two experienced neuroradiologists visually inspected the DTI images to screen for noise artifacts. The image data were analyzed using the FMRIB software library (FSL, version 4.1.8; http://www.fmrib.ox.ac.uk/fsl). The *dcm2niigui* was used to convert the DICOM format of all the diffused and T1-weighted data into the NIFTI format. With the *topup* tool, distortions due to susceptibility were corrected by using anterior-to-posterior and posterior-to-anterior phase-encoding directions. Head motion and eddy current distortions were correct by *eddy_openmp* command. Brain masks from the *b0* image of each participant were created using FSL's *BET* (Brain Extraction Tool). Based on a diffusion tensor model, the FSL toolbox *DTIFIT* was used to fit the pre-processed image to generate the FA, MD, AD, and RD values. The different measures of the white matter micro-structure were extracted from diffusion images. The FA is a summary measure of micro-structural integrity of white matter. Diffusivity perpendicular to the axonal fibers, as measured by the RD, is strongly correlated with dysmyelination and demyelination processes. Diffusivity parallel to the axonal fibers is calculated by AD and the MD represents average diffusivity of molecular motion.

With the nonlinear registration tool of FNIRT (FMRIB’s Nonlinear Registration Tool), the FA, MD, AD, and RD images of each participant were registered to the FMRIB58_FA template in the Montreal Neurological Institute (MNI) space. By using FA threshold value of 0.2, a mean FA image was calculated from all the participants’ images and was thinned to create a mean FA skeleton. Each participant’s aligned FA map was projected onto the skeleton using the maximum FA values detected in the direction surrounding the tract. Finally, the AD, RD and MD skeleton were created by using the projection parameters obtained in the previous steps. Because TBSS only analyzed the voxels closest to the white matter fiber skeleton, it can effectively reduce statistical errors caused by imperfect registration. In addition, the non-parametric test was used in the statistical analysis, which did not require image smoothing; thus, the partial volume problem had been reduced.

### Statistical analysis

The sample size was based on a cross-sectional study design and the FA values of the white matter tracts in the NDPH and HC groups. A sample size of 46 (25 HCs and 21 patients with NDPH) achieved 86% power to reject the null hypothesis of equal means when the mean difference was 0.025, with a standard deviation for both groups of 0.027 at a two-sided alpha of 0.05. Stata 16.0 software (StataCorp LLC, TX, USA) was used for all clinical data analyses. Age, BMI, and other continuous variables were reported as mean ± standard deviation (SD) or median (interquartile range) and analyzed using independent sample t-tests or Mann–Whitney tests, respectively. Differences between genders were compared using the chi-square test. Statistical significance was set at *p* < 0.05. We executed voxel-wise cross-subject statistics of two-sample t-tests between patients with NDPH and HCs, which was carried out by FSL Randomize tool (version 2.1) using 5,000 permutations with the threshold-free cluster enhancement (TFCE) option. Although age and gender did not show significant differences between groups, they were added as covariates to minimize their potential effects. Multiple comparison correction was carried out with the family wise error (FWE) method applying the TFCE option in the FSL permutation-based inference tool by nonparametric statistics called “randomise”. Statistical maps were set at *p* < 0.05 (two-sided, FWE corrected) with a cluster size of > 100 voxels [13]. Pearson’s correlation analysis was conducted to examine correlations between FA, MD, AD, and RD values and the clinical characteristics (disease duration of NDPH [years], VAS scores, HIT-6 scores, PHQ-9 scores, GAD-7 scores, and PSQI scores). The level of statistical significance was set at *p* < 0.05/N (Bonferroni-corrected for multiple comparisons; N corresponds to the number of correlation analyses tested).

## Results

### Demographics and clinical characteristics

**Fig. 1:**
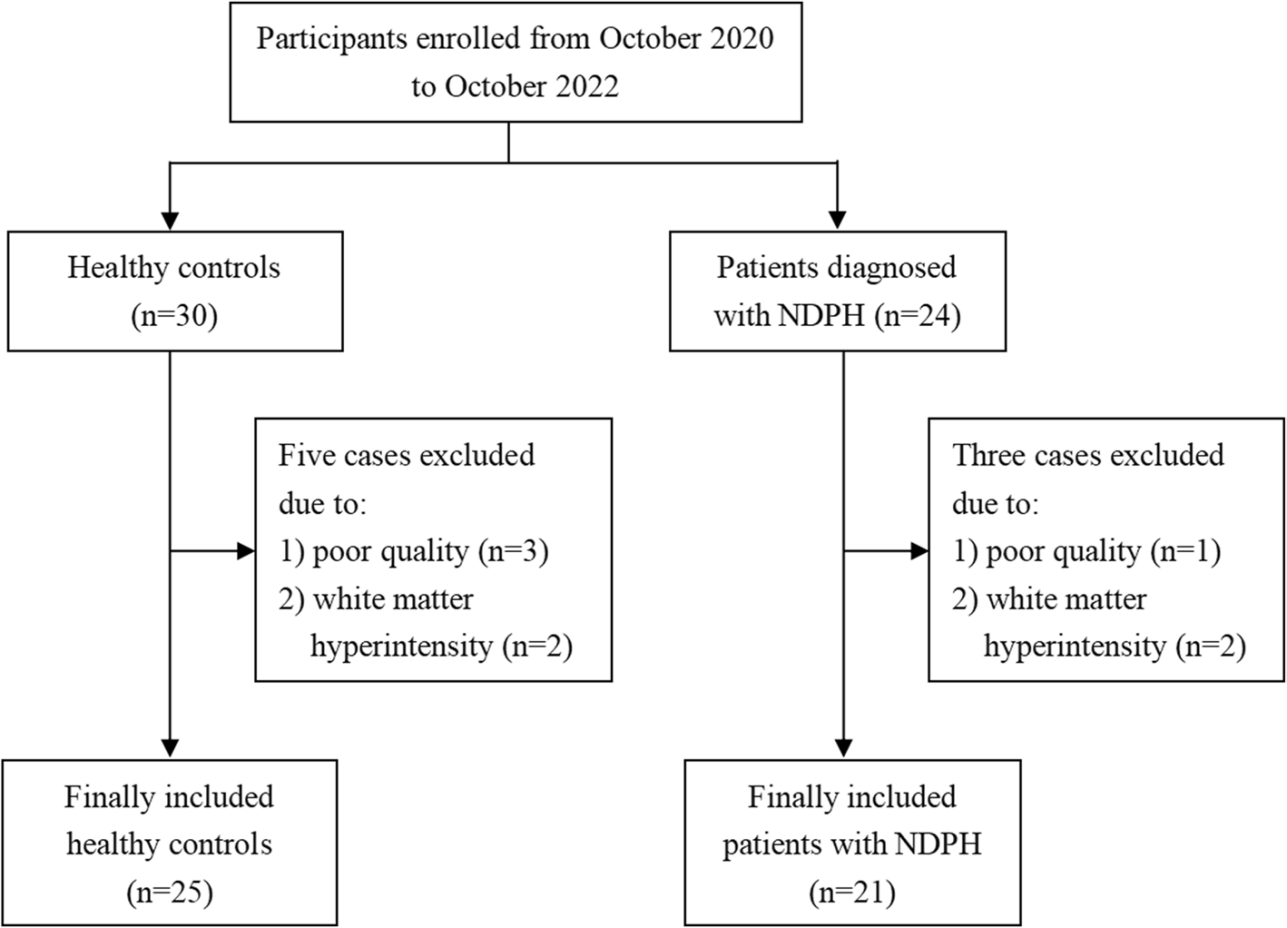
Flowchart of the participant inclusion process. Note: NDPH, new daily persistent headache

Demographic and clinical data of the enrolled participants were presented in Table 1. We recruited 54 participants with 24 patients with NDPH and 30 HCs in our initial cohort. Three participants with NDPH were excluded owing to the poor quality of the MRI data (*n* = 1) and existing white matter hyperintensities (*n* = 2). Five HCs were also excluded because of the poor quality of the MRI data (*n* = 3) and existing white matter hyperintensities (*n* = 2) (Fig. 1). Finally, a total of 46 participants (21 patients with NDPH and 25 HCs) were included, and no differences in age, gender ratio, or BMI were found between the two groups (Table 1).

**Table 1:** Demographic and clinical data

	Controls (*n* = 25)	NDPH (*n* = 21)	*P* value
Ages, years	30.60 ± 7.37	28.81 ± 15.1	0.30
BMI	22.00 ± 3.14	25.05 ± 4.16	0.06
Gender (female/male)	10/15	14/7	0.07
Headache history (years)	/	9.31 ± 11.39	/
Pain intensity VAS score	/	4.76 ± 2.10	/
HIT-6 score	/	61.78 ± 11.90	/
PHQ-9 score	/	10.11 ± 7.62	/
GAD-7 score	/	7.52 ± 5.61	/
PSQI score	/	9.00 ± 4.97	/

*Note*: *NDPH* new daily persistent headache, *BMI* body mass index, *VAS* Visual Analogue Scale, *HIT-6* Headache Impact Test-6, *PHQ-9* Patient Health Questionnaire-9, *GAD-7* Generalized Anxiety Disorder-7, *PSQI* Pittsburgh Sleep Quality Index

### TBSS analysis of FA

**Fig. 2:**
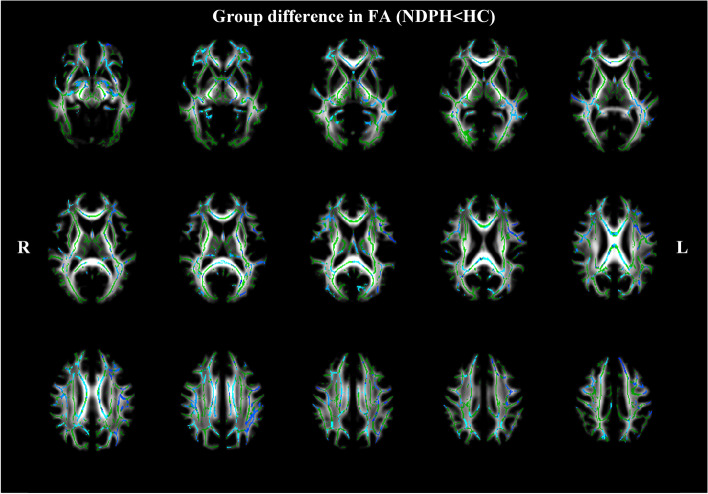
White matter regions (blue) showed decreased fractional anisotropy (FA) values in patients with NDPH compared to HC group (*P*_FWE_ < 0.05). The white matter regions with green color represented FA skeleton. Note: NDPH, new daily persistent headache; HC: healthy control; L: left; R: right

Compared to HCs, the NDPH group showed significantly decreased FA in several white matter tracts (*P*_FWE_ < 0.05). These white matter tracts included the bilateral corona radiata (CR), left anterior limb of the internal capsule (ALIC), bilateral anterior thalamic radiation (ATR), corpus callosum (CC), bilateral cingulum, bilateral external capsule (EC), fornix, forceps minor, bilateral inferior fronto-occipital fasciculus (IFOF), bilateral inferior longitudinal fasciculus (ILF), bilateral posterior thalamic radiation (PTR), bilateral retrolenticular part of the internal capsule (RPIC), and bilateral superior longitudinal fasciculus (SLF) (Fig. 2) (*P*_FWE_ < 0.05).

### TBSS analysis of MD

**Fig. 3:**
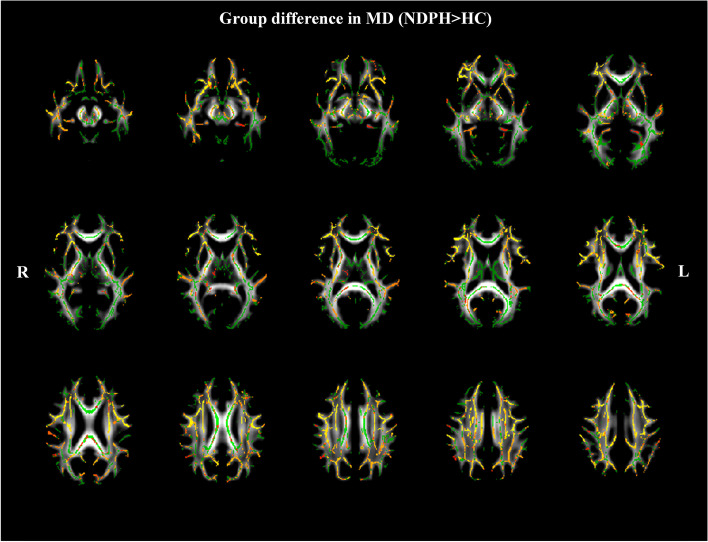
White matter regions (warm color: yellow–red) showed increased mean diffusivity (MD) values in patients with NDPH compared to HC group (*P*_FWE_ < 0.05). The white matter regions with green color represented MD skeleton. Note: NDPH, new daily persistent headache; HC: healthy control; L: left; R: right

Compared to HCs, patients with NDPH presented with significantly higher MD in several white matter tracts. These white matter tracts included the bilateral CR, bilateral ALIC, bilateral ATR, CC, bilateral cerebral peduncle (CP), bilateral corticospinal tract (CST), cingulum, bilateral EC, forceps minor, forceps major, fornix, bilateral IFOF, bilateral ILF, bilateral posterior limb of the internal capsule (PLIC), right PTR, right RPIC, bilateral SLF, and bilateral uncinate fasciculus (UF) (Fig. 3) (*P*_FWE_ < 0.05).

### TBSS analysis of RD

**Fig. 4:**
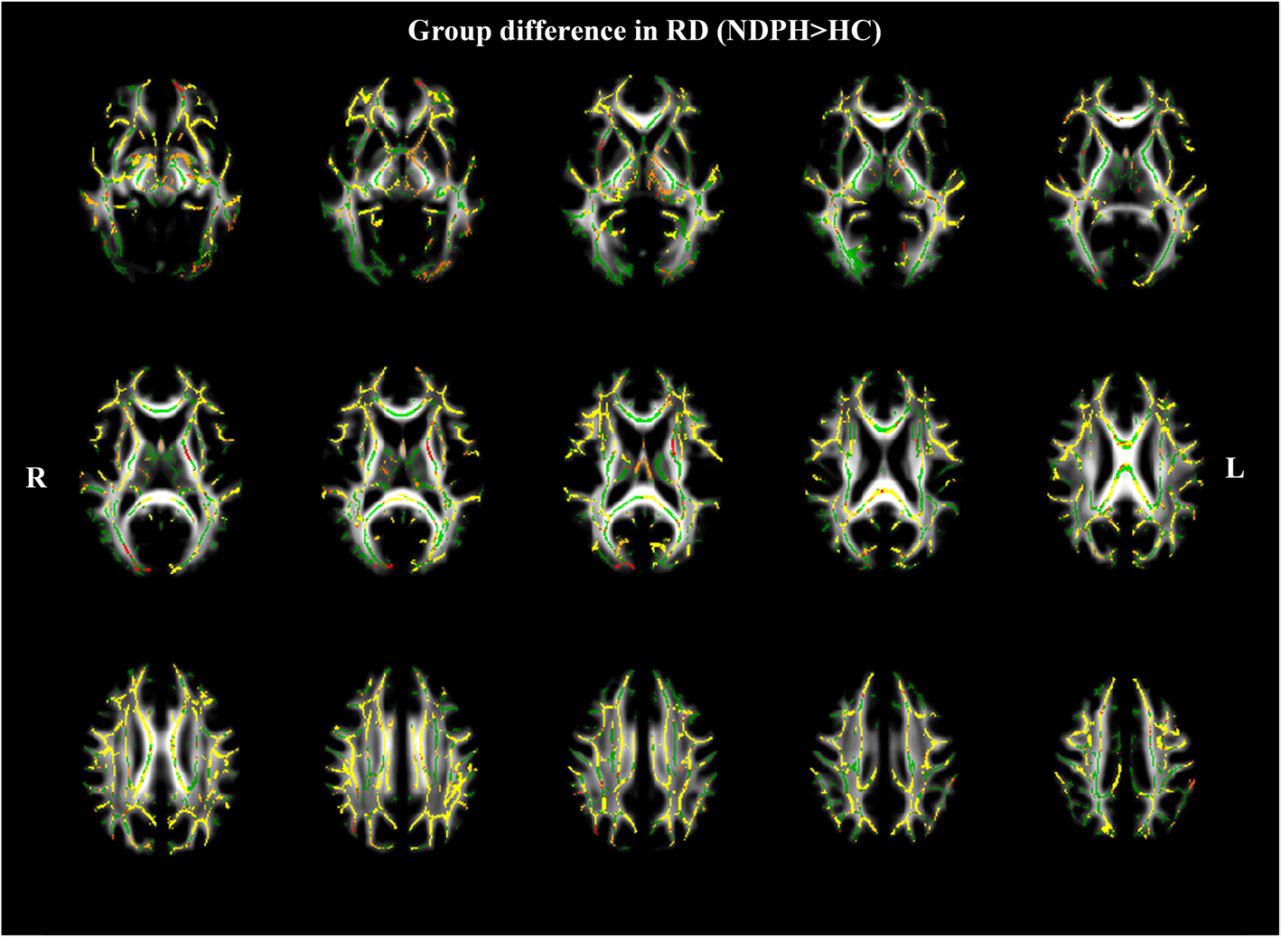
White matter regions (warm color: yellow–red) showed increased radial diffusion (RD) values in patients with NDPH compared to HC group (*P*_FWE_ < 0.05). The white matter regions with green color represented RD skeleton. Note: NDPH, new daily persistent headache; HC: healthy control; L: left; R: right

Compared to the HCs, patients in the NDPH group exhibited higher RD in several brain regions. These regions included the bilateral CR, bilateral ALIC, bilateral ATR, right CST, CC, cingulum, bilateral EC, forceps minor, forceps major, fornix, genu of the corpus callosum (GCC), bilateral IFOF, bilateral ILF, left PLIC, bilateral PTR, bilateral RPIC, splenium of the corpus callosum (SCC), bilateral superior CR, bilateral SLF, and left UF (Fig. 4) (*P*_FWE_ < 0.05).

### TBSS analysis of overlapping maps of FA, MD and RD

**Fig. 5:**
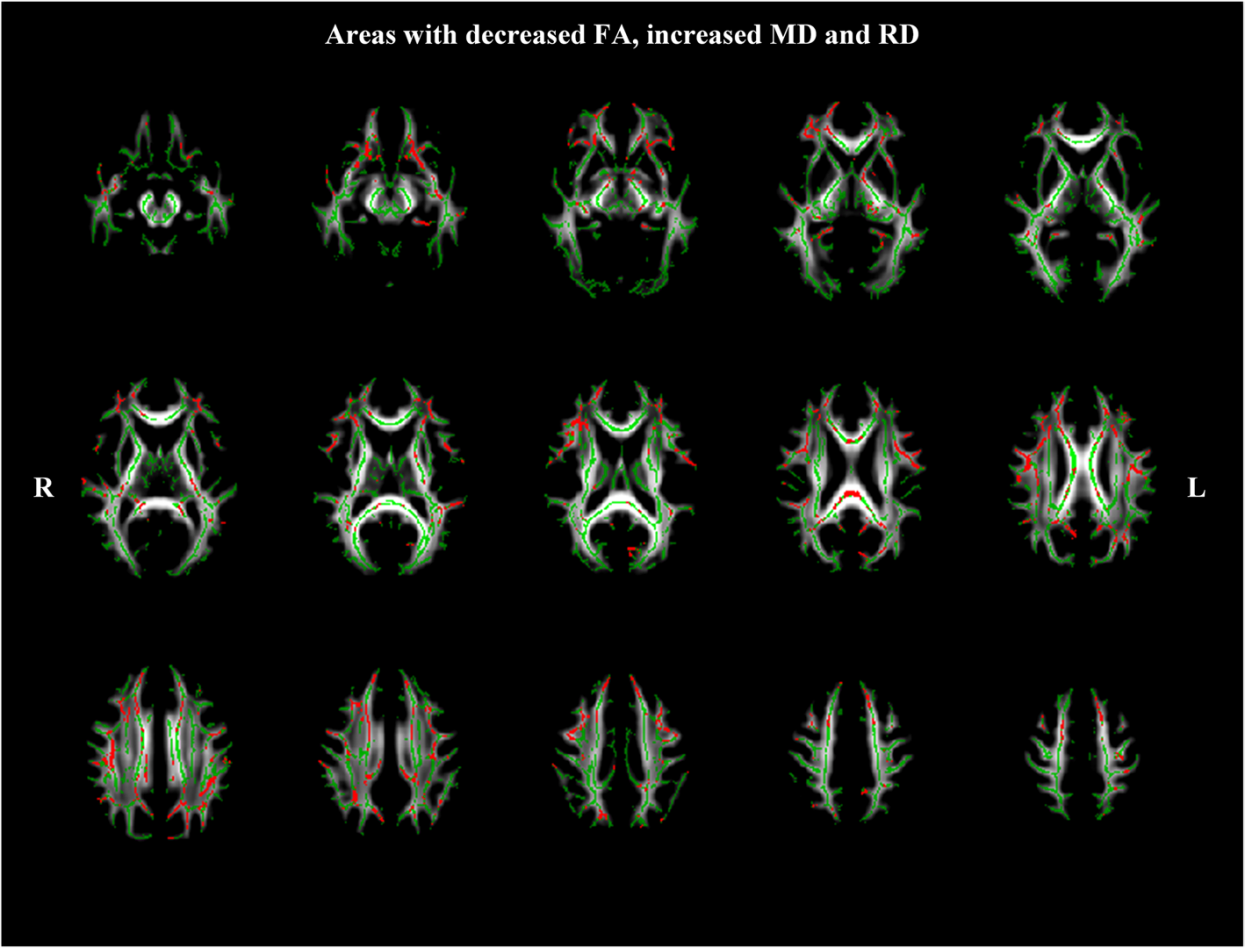
White matter regions (red color) showed decreased FA, increased RD and MD values in patients with NDPH compared to HC group (*P*_FWE_ < 0.05). The white matter regions with green color represented FA skeleton. Note: NDPH, new daily persistent headache; HC: healthy control; L: left; R: right

As shown in the Fig. 5, the overlap of the decrease of FA with increase of MD and RD in patients with NDPH group compared to HCs group was shown in red, which included the right ATR, body of the corpus callosum (BCC), bilateral cingulum, left hippocampal cingulum (CGH), left CST, forceps major, fornix, left IFOF, bilateral ILF, left PLIC, right RPIC, SCC, right SLF, and left UF (*P*_FWE_ < 0.05).

### The correlations between FA of overlapping tracts and clinical characteristics in patients with NDPH

For Pearson’s correlation analysis, we selected the regions showing overlapping differences in FA, MD and RD values between patients with NDPH and HCs (Table 2). The FA, MD, AD, and RD values of these regions were extracted. There were no significant correlations between the FA, MD, AD, and RD values of the overlapping tracts and the clinical characteristics (pain intensity, disease duration, HIT-6 score, GAD-7 score, PHQ-9 score, and PSQI score) after Bonferroni correction (*p* > 0.05/96).

**Table 2:** Whiter matter with decreased FA, increased MD and RD (*P*_FWE_ < 0.05)

Cluster size (Voxels)	MNI center of mass			Side	Brain region	Related function
	X	Y	Z			
2194	-28	45	1	L	IFOF	Language, emotion
1189	-25	-72	18	/	Forceps major	Emotion, pain processing
734	9	-51	7	R	Cingulum	Motivation, pain and emotion interactions
440	47	-53	26	R	SLF	Cognition, emotion
422	43	-9	-27	R	ILF	Language, visual memory, emotion
414	-12	-46	26	L	Cingulum	Motivation, emotion, pain perception
393	-6	15	20	/	BCC	Pain processing, emotion, cognition
389	-41	-11	-18	L	ILF	Language, visual memory, emotion
384	-23	-32	-15	L	CGH	Emotion, cognition
248	33	-15	-11	/	Fornix	Memory, emotion
229	-34	1	-13	L	UF	Emotion, cognition, language
148	10	-8	-12	R	ATR	Emotion
145	18	-40	31	/	SCC	Pain processing, emotion, cognition
135	28	-21	1	R	RPIC	Visual sense
124	-16	-23	-8	L	CST	Motor
113	-18	-7	8	L	PLIC	Pain processing

*Note*: *MNI* Montreal Neurological Institute, *L* left, *R* right, *IFOF* inferior fronto-occipital fasciculus, *SLF* superior longitudinal fasciculus, *ILF* inferior longitudinal fasciculus, *BCC* body of corpus callosum, *CGH* hippocampal cingulum, *UF* uncinate fasciculus, *ATR* anterior thalamic radiation, *SCC* splenium of corpus callosum, *RPIC* retrolenticular part of internal capsule, *CST* corticospinal tract, *PLIC* posterior limb of internal capsule

## Discussion

Our study first performed TBSS analysis to investigate abnormalities in white matter micro-structure in patients with NDPH. The initial results revealed that patients with NDPH exhibited significantly decreased FA, increased MD and RD compared to HCs. White matter regions overlaid with decreased FA, increased MD and RD were found in the right ATR, BCC, bilateral cingulum, left CGH, left CST, forceps major, fornix, left IFOF, bilateral ILF, left PLIC, right RPIC, SCC, right SLF, and left UF (Table 2).

Compared with voxel-based analysis method in DTI [28, 29], TBSS method is better in image alignment and without the smoothing problem [30, 31]. Several DTI-derived metrics based on TBSS, including FA, MD, AD, and RD, are sensitive to changes in white matter micro-structural. A low FA values may be caused by several conditions, including axonal loss, inflammation, demyelination, and gliosis [18]. The MD is an inverse measure of the membrane density and reflects both cellular swelling and cellular density [32]. AD is a measure of diffusivity parallel to axonal fibers, and a reduced AD may indicate axonal loss [18]. The RD increases in white matter with disrupted myelination [19]. Changes in the axonal diameters or density may also influence RD.

Given that FA is a summary measure of micro-structural integrity, MD and RD represent inverse measures of the membrane density and white matter with de- or dys-myelination [19]. Our findings may reveal micro-structural white matter abnormalities, especially disrupted myelination or decreased cellular density (such as gliosis) in patients with NDPH compared with HCs (shown in red in Fig. 5). Since the activation of glial cells and neuro-glial interactions may be key mechanism underlying chronic pain [33], our results may suggest that white matter abnormalities in this study may be connected with the chronic and unremitting pain state in patients with NDPH. Moreover, the white matter tracts that exhibited abnormal diffusive metrics in this TBSS study are mainly involved with the transmission and integration of sensory, cognitive, and/or emotional information. Consistent with previous findings of DTI study in other primary headache, especially migraine, we can firmly believe that the diffuse white matter micro-structural changes can disrupt the pain perception and lead to chronic pain [11–14].

The IFOF and UF are primarily associated with functions related to language and emotion [34, 35], which might potentially be relevant in the context of chronic pain experience in NDPH. The IFOF courses between the frontal and occipital lobes and may be involved in the language system and provide anatomic connections for visuospatial attention [34]. Meanwhile, the UF connects parts of the limbic system (temporal pole, amygdala, and anterior parahippocampus) with inferior portions of the frontal lobe (orbitofrontal cortex) [35]. The UF links emotion and cognition to the ventral limbic pathway [36]. Evidence from previous researches suggested that these tracts were involved in several cognitive and emotional processes, such as semantic processing of language, auditory working memory, and sound recognition [37]. Furthermore, studies have found micro-structural white matter abnormalities in these regions to be associated with major depressive disorder and other psychiatric disorders [38–40]. However, it is crucial to clarify that the patients with NDPH in this study did not have any pre-existing psychiatric disorders before the onset of their headaches. Some patients with NDPH developed symptoms of depression and/or anxiety as a result of enduring persistent headaches. Interestingly, Chen et al. have proposed that a reduction in myelin density in the IFOF and UF might contribute to the pathophysiology of major depressive disorder (MDD) [41]. Although distinct differences exist between MDD and NDPH, these findings underscore the possibility that micro-structural abnormalities in the white matter of the IFOF and UF may also be relevant to the neurobiology of NDPH. In the context of NDPH, it would be speculative, but interesting, to consider whether these micro-structural changes in the IFOF and UF could be linked to the chronic pain experience. Future studies investigating the potential associations between these white matter changes and specific symptom domains in patients with NDPH could provide valuable insights. For example, comparing NDPH patients with and without comorbid affective symptoms might elucidate whether these tract changes are more pronounced in patients with additional emotional distress.

The current study revealed the significant involvement of the CC, forceps major, cingulum, and PLIC in NDPH, notably their roles in the pain processing network. The CC, as a key interhemispheric commissural pathway, connects most neocortical areas and plays a central role in the integration of perceptual, cognitive, and volitional information across the hemispheres. The callosal fibers arising from the SCC that interconnect the parieto-occipital regions are called the forceps major. The fibers projecting into the primary somatosensory cortex are contained in the BCC. The SCC is the most posterior part of the corpus callosum, which connects somatosensory perception between the parietal lobe of the two hemispheres and the visual center in the occipital lobe [42]. Lots of experimental and clinical studies [43–45] have found that the CC and its components, including the forceps major, are involved in pain modulation and processing within the central nervous system. The cingulum, a core part of the limbic system, has been implicated in several brain functions, including pain perception [46, 47]. In this study, we found micro-structural white matter abnormalities in the left PLIC. The PLIC contains projection fibers of facial and somatosensory signals that arrive at the thalamus. Given the critical role of the PLIC, such abnormalities could be crucial for understanding the pain processing mechanisms in NDPH. Moreover, some previous studies have reported patients with primary headache, such as migraine and cluster headache showed micro-structure changes in various white matter regions, including the CC, forceps major and minor, cingulum, and internal and external capsules [13, 14, 48]. This finding further corroborates the notion that these regions could be integral to the pathophysiology of headache disorders, including NDPH. In summary, the CC, cingulum, and internal capsule (IC) play indispensable roles in pain processing in patients with NDPH. Further studies are needed to explore the correlation between white matter abnormalities in these regions and NDPH symptomatology. This could help illuminate the shared pathophysiological mechanisms across headache and painful disorders, and guide future therapeutic approaches.

As a part of the limbic system, the fornix originates from the hippocampus and stretches to the diencephalon and basal forebrain. Although the fornix itself is not primarily associated with pain perception, it plays a crucial role in cognitive processes, such as memory, learning, and emotional response, which can significantly influence the perception and processing of pain [49]. The SLF is the main long association fiber pathway in the suprasylvian area. The temporal pole and the dorsolateral occipital lobe are connected by the ILF, which lies within the inferior temporal lobe [50]. Of significant note, Rahimi et al. documented alterations in the white matter of these tracts in patients with migraine [51]. This suggests that the SLF and ILF might contribute to the neuropathology of migraines, perhaps by influencing the perception and processing of pain associated with these conditions. Further investigation is required to clarify the extent and implications of these changes in the context of headache disorders, such as NDPH.

Previous TBSS studies on migraine and cluster headache have also played an important role in exploring the pathophysiological changes in primary headache. These findings demonstrated alterations in FA, MD, AD, and RD across these types of headache. Specifically, there were decreased FA, MD and AD values in several white matter regions in patients with episodic migraine compared to the HCs [14]. In addition, the RD and MD values of white matter were significantly higher in chronic migraine than in HCs [13]. Furthermore, the decreased FA values and the increased AD, MD, and RD values were found in patients with cluster headache compared with the HCs [48]. However, significantly decreased FA, increased MD and RD were found in patients with NDPH compared to HCs. The above studies indicated that the pattern of white matter micro-structural changes in patients with NDPH was slightly different from that in patients with other types of primary headaches. Notably, while previous studies have reported associations between white matter hyperintensities or increased risk for ischemic stroke and migraine [52, 53], such hyperintensities are absent in NDPH. This absence further points to distinctive white matter changes in NDPH compared to other primary headaches, and warrants further exploration for a comprehensive understanding of these neurological conditions.

A few limitations of the study should be noted. First, this study was a cross-sectional design research, so it was hard to draw causal conclusions. Second, the study's small sample size might have biased our results and our preliminary results need to be verified with more data in the future. Third, there were few adolescents in the NDPH group. However, our control group did not include adolescents due to the practical limitations of the study. Fourth, psychotic comorbidities, especially depression and anxiety, could affect brain structure even at low grade and untreated levels. Further research is needed to determine whether this type of comorbidities may cause the micro-structural abnormalities detected in this study.

## Conclusion

Our results suggested that patients with NDPH can be related to the widespread white matter abnormalities in the brain. Significantly decreased FA, increased MD and RD were found in patients with NDPH compared to HCs in 16 white matter regions from the Johns Hopkins University ICBM-DTI-81 White-Matter Atlas and Johns Hopkins University White-Matter Tractography Atlas. Furthermore, the subtle structural changes in white matter were primarily involved in emotion, cognition and pain modulation. In addition, our TBSS results suggested disrupted myelination or decreased cellular density in the above white matter regions of patients with NDPH. However, more comprehensive experimental designs are required to determine the exact role of these white matter changes in pathology of NDPH. We hope that our results can improve the understanding of the mechanism of NDPH and provide therapeutic strategies and potential diagnostic information for patients with NDPH.

## Data Availability

Data can be made available upon request.

## Abbreviations

AD: Axial diffusivity
ALIC: Anterior limb of internal capsule
ATR: Anterior thalamic radiation
BCC: Body of corpus callosum
BMI: Body mass index
CC: Corpus callosum
CGH: Hippocampal cingulum
CP: Cerebral peduncle
CR: Corona radiata
CST: Corticospinal tract
DTI: Diffusion tensor imaging
EC: External capsule
FA: Fractional anisotropy
FSL: FMRIB software library
FWE: Family wise error
GAD-7: Generalized anxiety disorder-7
GCC: Genu of corpus callosum
HCs: Healthy controls
HIT-6: Headache Impact Test-6
IC: Internal capsule
ICHD-3: International classification of headache diseases, 3rd edition
IFOF: Inferior fronto-occipital fasciculus
ILF: Inferior longitudinal fasciculus
MD: Mean diffusivity
MDD: Major depressive disorder
MNI: Montreal neurological Institute
MRI: Magnetic resonance imaging
NDPH: New daily persistent headache
PHQ-9: Patient health questionnaire-9
PLIC: Posterior limb of internal capsule
PSQI: Pittsburgh sleep quality index
PTR: Posterior thalamic radiation
RD: Radial diffusivity
RPIC: Retrolenticular part of internal capsule
SCC: Splenium of corpus callosum
SD: Standard deviation
SLF: Superior longitudinal fasciculus
TBSS: Tract-based spatial statistics
TFCE: Threshold-free cluster enhancement
UF: Uncinate fasciculus
VAS: Visual analogue scale
